# Supplementary material for: Splice-Junction-Based Mapping of Alternative Isoforms in the Human Proteome
Source: Cell Rep. Author manuscript; Available in PMC 2020 Jan 15. (PMC6961840; doi:10.1016/j.celrep.2019.11.026)
Supplement: 2 [file NIHMS1546469-supplement-2.zip › DF1/PXD010154/Lung/Lung_42_LLGL2_VEVPEAMTPLSLPVQER.pdf]

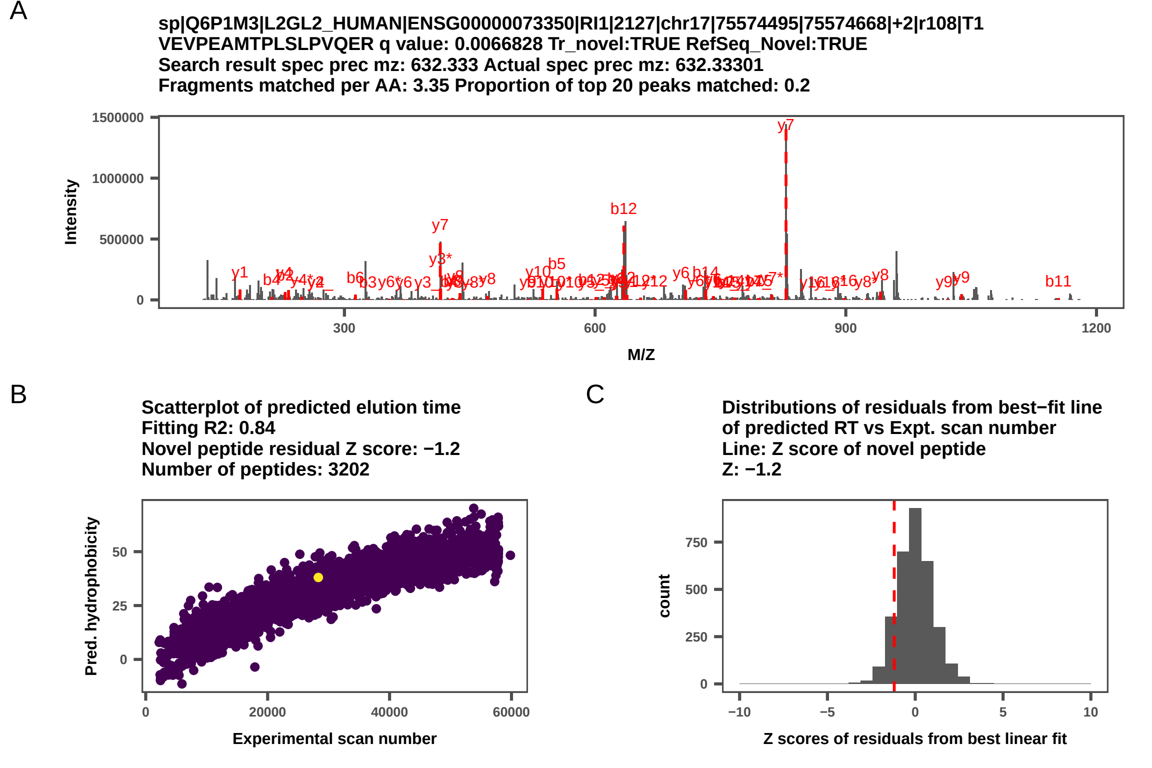
A
sp|Q6P1M3|L2GL2_HUMAN|ENSG00000073350|RI1|2127|chr17|75574495|75574668|+2|r108|T1
VEVPEAMTPLSLPVQER q value: 0.0066828 Tr_novel:TRUE RefSeq_Novel:TRUE
Search result spec prec mz: 632.333 Actual spec prec mz: 632.33301
Fragments matched per AA: 3.35 Proportion of top 20 peaks matched: 0.2
Intensity
1500000
1000000
500000
0
y7
b12
y7
y3*
y1
b5
b11
300
600
900
1200
M/Z
B
Scatterplot of predicted elution time
Fitting R2: 0.84
Novel peptide residual Z score: −1.2
Number of peptides: 3202
Pred. hydrophobicity
50
25
0
0
20000
40000
60000
Experimental scan number
C
Distributions of residuals from best−fit line
of predicted RT vs Expt. scan number
Line: Z score of novel peptide
Z: −1.2
count
750
500
250
0
−10
−5
0
5
10
Z scores of residuals from best linear fit
